# P-2114. Pattern of Clinical Characteristics, Outcome and Comparison of Carbapenem Resistant and Carbapenem Sensitive *Klebsiella Species* Infections from a Tertiary Care Hospital, Karachi, Pakistan

**DOI:** 10.1093/ofid/ofae631.2270

**Published:** 2025-01-29

**Authors:** Rohama Samar, Mohammad Kashif Farooq, Zaheer Udin Babar, Sunil Dodani

**Affiliations:** Sindh Institute of Urology and Transplantation, Karachi, Sindh, Pakistan; Sindh Institute of Urology and Transplantation, Karachi, Sindh, Pakistan; Sindh Institute of Urology and Transplantation, Karachi, Sindh, Pakistan; Sindh Institute of Urology and Transplantation, Karachi, Sindh, Pakistan

## Abstract

**Background:**

The prevalence of carbapenem resistant (CR) Klebsiella species (KS) is greater than 60 % in South Asia with high morbidity and mortality. Data on clinical aspects of KS from Pakistan is scarce. We aimed to study clinical characteristics, outcome and risk factors for mortality among KS infection

**Figure:**
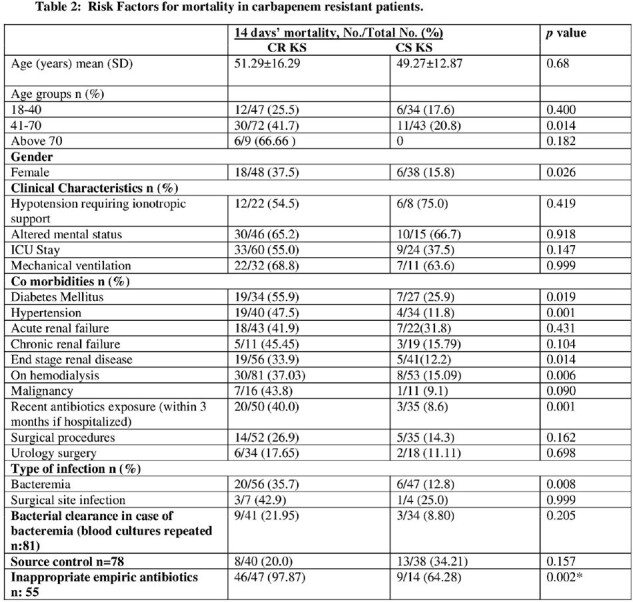

**Methods:**

A prospective study conducted from September 2022 to March 2023. All patients greater than 18 years with active KS infection were included. Age, gender, clinical features, Charlson co-morbidity index, source of infection, previous hospitalization, source control and appropriate empirical antibiotics received were noted. Outcome was documented as admitted, discharged or death at day 14.

CRKS and carbapenem sensitive (CSKS) were compared.

**Table 1:**
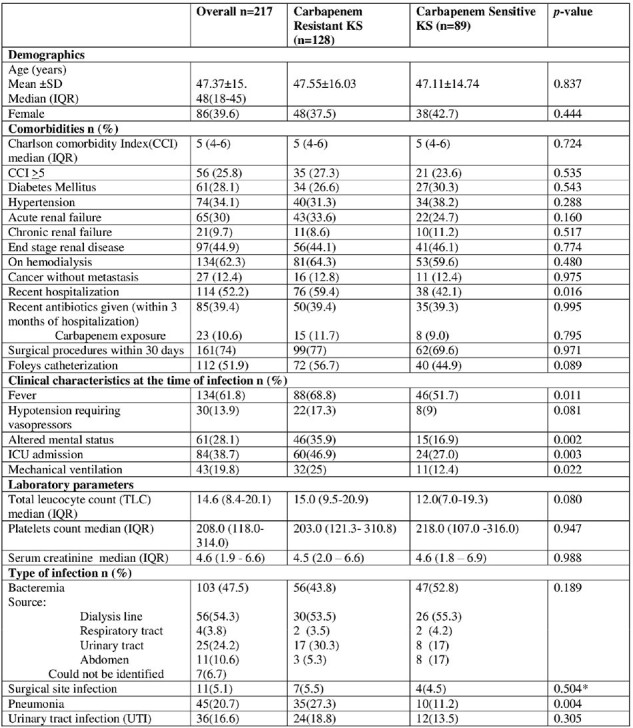
Demographics, Risk factors and outcome of CRKS and CSKS infection.

**Results:**

Out of 217, 128 (58.9%) CRKS and 89 (41%) CSKS infection. The median age was 48 years, 39.6% female. Around 114 (52%) had previous hospitalization. Bacteremia 103(47.5) was most common. At day 14, all-cause mortality was 29%. CRKS infection was significantly associated with recent hospitalization, fever, altered mental status, ICU admission and mechanical ventilation.

Pneumonia was more common with CRKS (p=value 0.004). Among CRKS, 4 (3.2%) received appropriate empirical antibiotics and had poor bacterial clearance (p=0.048). A survival curve shows significant high mortality in CRKS over 14 days. On multivariate analysis patients with CRKS did not receive appropriate empirical antibiotics.

**Table 1:**
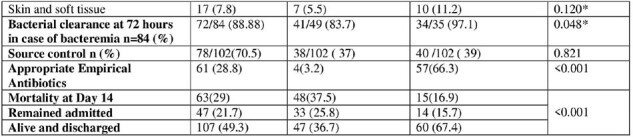
Demographics, Risk factors and outcome of CRKS and CSKS infection.

**Conclusion:**

We found a very high proportion of CRKS infection with high morbidity and mortality. Patients with CRKS infection did not receive appropriate empirical antibiotics. Timely availability of antibiogram with good infection control practices are critical in preventing mortality and spread this highly pathogenic organism.

**Figure:**
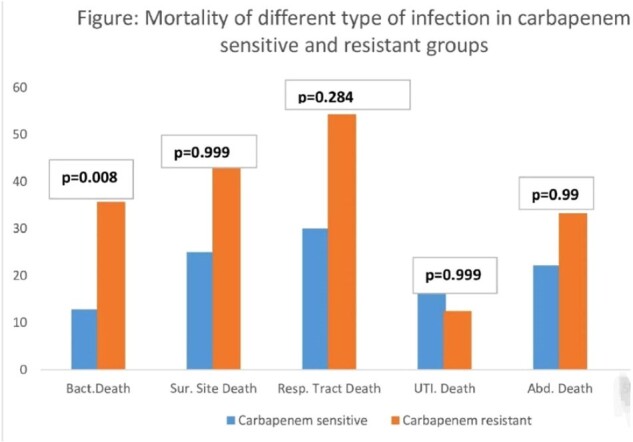

**Disclosures:**

All Authors: No reported disclosures

